# eDNA metabarcoding reveals high soil fungal diversity and variation in community composition among Spanish cliffs

**DOI:** 10.1002/ece3.9594

**Published:** 2022-12-12

**Authors:** Franz‐Sebastian Krah, Martí March‐Salas

**Affiliations:** ^1^ Faculty of Biological Sciences, Institute for Ecology, Evolution and Diversity, Conservation Biology Goethe University Frankfurt Frankfurt am Main Germany; ^2^ Faculty of Biological Sciences, Institute for Ecology, Evolution and Diversity, Plant Evolutionary Ecology Goethe University Frankfurt Frankfurt am Main Germany

**Keywords:** cliff diversity, eDNA metabarcoding, extreme environments, fungal community composition, rock‐specialist plant species, spatial biodiversity variations

## Abstract

Environments characterized by physical extremes harbor unique species diversity with particular adaptations. Cliffs are harsh environments for organisms but host a great diversity of specialized plants with many endemics, rare and even endangered species. It is, however, less known which fungal diversity the cliff habitats contain and whether it differs among different cliff locations. We thus sampled soil from three separate cliff locations in the North, Centre, and South of Spain and used eDNA metabarcoding to determine fungal diversity. To better understand whether cliff specialist plants may promote particular fungal communities, we have sampled soil from crevices with cliff specialist plants and no apparent plants as controls. Major lifestyles found in cliff soils were saprotrophs, and major fungal orders were Dothideomycetes, Sordariomycetes, and Eurotiomycetes, while the amount of symbiotrophic fungi was relatively low. We found no significant differences in fungal amplicon sequence variant (ASV) richness among the three sampled locations, but the sites were significantly different in their community composition and their main indicator species. Overall, there were no significant differences in fungal ASV richness or composition between soils from cliff specialist plants and soils without plants, suggesting a unique fungal diversity in cliff soils independent from specialized plants. However, preliminary findings on soils of the specialist cliff plant *Sedum dasyphyllum* against control soils suggest that the presence of a specialist plant may be a relevant factor affecting the specificity of the fungal community in cliff soils. Our results indicate the existence of particular cliff fungal communities in each location, and that, despite limited and poorly developed soils and harsh conditions, cliffs can harbor a great diversity of fungal species, comparable to other ecosystems of Spain. This study points out that some fungi may be cliff‐specific, shaping particular communities that mediate plant adaptations to cliffs' extreme conditions.

## Graphical abstract

Cliffs are harsh environments that host specialized plants; however, the cliff fungal diversity is largely unknown. Here, we sequenced cliff soils for fungi in three locations in Spain and find composition differences between locations suggesting a variety of fungal strategies to cope with the harsh environments of cliffs.

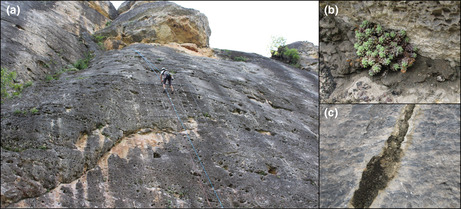

## 1 INTRODUCTION

Human activities affect ecosystems, leading to a global species loss (Newbold et al., 2015). This is especially true in extreme environments, which are at high risk due to their rarity and particular biodiversity (Ram & Gupta, 1997). Cliffs are an example of an extreme environment, ideal for studying species' adaptations to harsh environments considering drought, resource limitations, or low soil accumulation (Larson et al., 2000; March‐Salas et al., 2018). Besides habitat loss due to its high erosion or climate change, recreational activities such as rock climbing can affect the high biodiversity inhabiting cliffs (Burgin & Hardiman, 2012; Watson et al., 2014). Despite the distinguished physical features of cliff habitats and their recognized plant diversity, we currently have only limited information on fungal diversity in such environments.

Cliffs are characterized by harsh conditions, such as water limitation, poorly developed soils, and intense solar radiation (Larson et al., 2000). Despite these extreme conditions, cliff habitats provide a high diversity of microhabitats, harboring many plant species, often including endemic and endangered species (Larson et al., 2000; Lavergne et al., 2003). Particularly, Spain has been chosen as a key conservation territory and is one of the most biodiverse countries in the European Union, holding a great diversity of cliff specialist plants with more than 150 being cliffs threatened plant species (deCastro‐Arrazola et al., 2021; National Biodiversity Strategy, 2020). Moreover, a previous study indicated the need for establishing protection priorities in Spanish cliff habitats but mainly considered as criteria endangered plants and natural protected areas (deCastro‐Arrazola et al., 2021). These protected areas are thus meant to protect rare and threatened plant and animal species and serve as fundamental tools for ecosystem management and conservation. Still, the presence of diverse fungal communities has been not considered in these assessments.

Fungi are an exceptionally species‐rich group with currently 146,000 described species (Bánki et al., 2022) and an estimated diversity of 1.5–3.8 million species (Hawksworth & Luecking, 2017). However, a recent review indicates that fungal microbial growth can be limited in extreme environments since, to survive in hostile habitats, fungi require investing considerable energy into cellular mechanisms (Gostinčar et al., 2022). By contrast, other studies found that their diversity can be high even in harsh and extreme conditions such as extreme deep‐sea environments (Nagano & Nagahama, 2012), extreme cold conditions (Gunde‐Cimerman et al., 2003), high salinity levels (Cantrell et al., 2011) or the Atacama desert (Santiago et al., 2018). Besides birds, insects, and plants, cliff environments can contain a rich microbial diversity, for example, algae, cyanobacteria, or bacteria (Gerrath et al., 2000; Horath & Bachofen, 2009), but also rock‐inhabiting fungi (Coleine et al., 2021). However, we currently have almost no knowledge of fungal communities in cliff soils (Cockell & Jones, 2009; Larson et al., 2000). Fungi may form mutualistic interactions with cliff plants, providing water and nutrients to the host plant and, in return, acquiring carbon from the plant (Kohler et al., 2015; Smith & Read, 2010). Saprotrophic fungi are the main actors in dead plant matter decomposition, mineralizing nutrients back into the system (Floudas et al., 2012). This may be functionally relevant for cliff plants, as they grow in small crevices. The generation of available nutrients could promote seedlings' emergence and recruitment or plant regrowth (in the case of perennials). Therefore, increasing knowledge of fungal communities in cliff habitats would help to comprehend the overall cliff diversity but also may be critical to evaluate their potential role in ecosystem functioning and plant's adaptation and specializations to these environments.

Fungi could also spatially differ in their richness and composition already at the ecosystem level (Branco et al., 2013). For example, previous research found latitudinal variation patterns of soil fungal community composition in Chinese forests (He et al., 2017). Mycorrhizal (symbiosis with plants) and saprotrophic (decomposer of dead organic matter) fungal community composition was also both significantly affected by varying soil moisture and temperature within Mediterranean pine forests (Castaño et al., 2018). At smaller scales, wood‐inhabiting fungal richness and community composition differed within the same forest type among tree species and between open and closed canopy stands (Krah et al., 2018). Therefore, even fine‐scale differences in fungal community composition across different sites on an ecosystem and regional scale may be possible, even considering a similar environmental context. However, extreme ecosystems can restrict the growth of some species since their harsh conditions are known to hinder fungal development (Gostinčar et al., 2022), so potential spatial variations in hostile ecosystems such as cliffs should be studied more profoundly.

**FIGURE 1:**
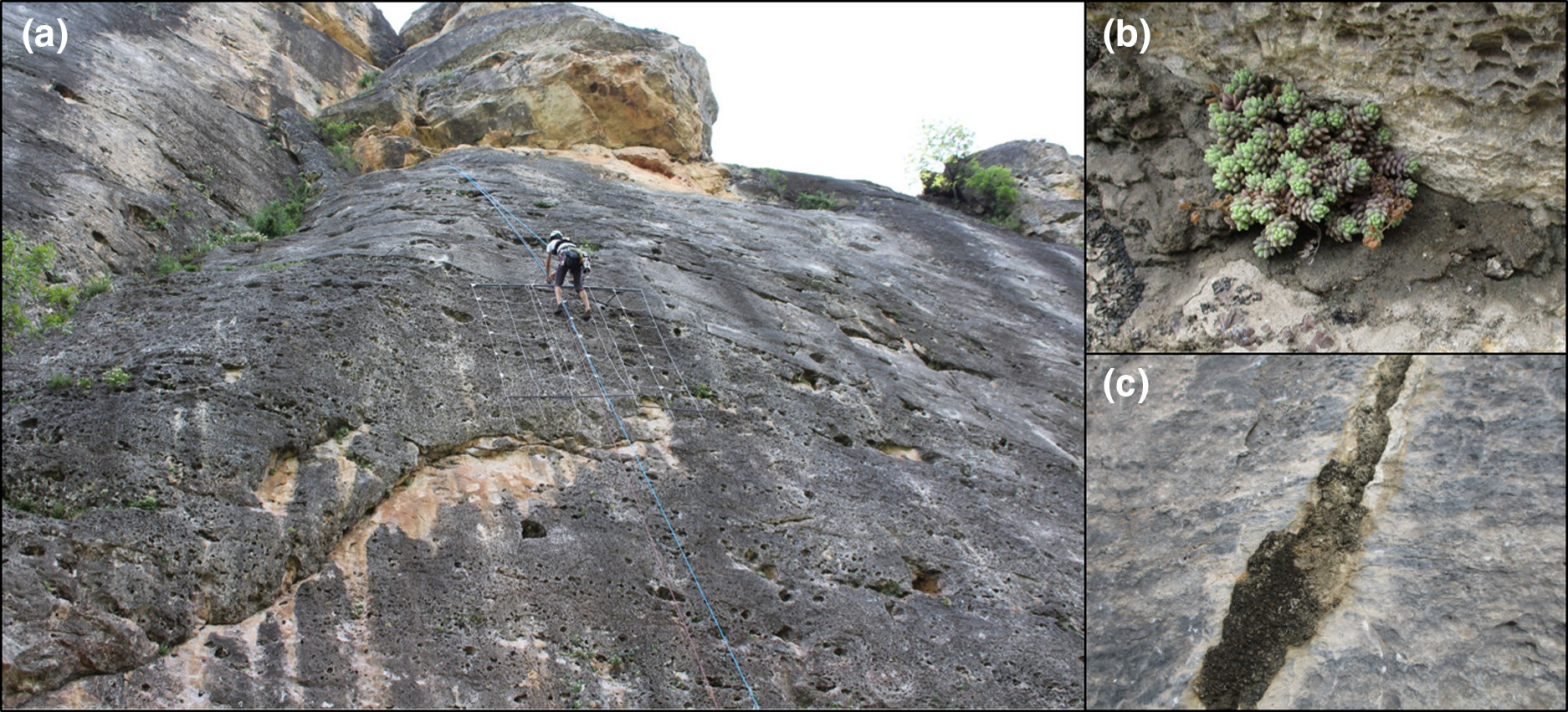
Example of cliff site and soil samples. (a) Sampled cliff in Patones (Madrid, Spain). To preclude samples from climbers' interference, the measures of a 3 × 3 m quadrat were indicatively used to guarantee the selection of soil samples more than 1 m far from the climbing line. (b) Individual of *Sedum dasyphyllum* and its surrounded soil, which was sampled for this study. (c) Crevice with soil with no apparent plant that was sampled for this study. Photographs by Martí March‐Salas.

In this study, we sampled soils inhabited by specialist cliff plants and soils without plants in limestone cliffs in three separate locations in Spain (North, Centre, and South of Iberian Peninsula, Table 1, Figure 1). We used high‐throughput amplicon sequencing of environmental DNA (eDNA) to assess fungal ASV (amplicon sequence variants) richness and composition in these samples. The main questions of this work are as follows: (1) What are the dominant trophic status and diversity of fungi in cliff soils? (2) Do fungi richness and composition differ among cliff locations? (3) Do cliff specialist plants promote differential soil fungal communities than soils without plants? To address these research questions, we quantify as alpha diversity the estimated number of ASVs and as beta diversity the community dissimilarity, all in it potentially helping us to determine the presence and distribution of fungi on cliff ecosystems along Spain.

**TABLE 1:** Summary information on study sites.

Location	Calcena	Patones Pontón de Oliva	Los Vados
Sampling date	10.05.2021	21.04.2021	19.04.2021
Zone	North	Centre	South
Area	Aragón (Spain)	Community of Madrid (Spain)	Andalucia (Spain)
City	Zaragoza	Madrid	Granada
Latitude	41.65	40.89	36.79
Longitude	−1.72	−3.44	−3.54
Altitude (m.N.N)	840	790	170
Rock type	Limestone		
Routes	Clásica, On the left to espolón, Los Gaiteros	Anubis, Guerra del grado, Vela verde	Three on the left, Po, Supernany
Annual Mean Temperature	11.5°C	18.0°C	17.3°C
Annual Precipitation	482 mm	276 mm	416 mm
Exposition	East	West	South‐East
Dominant vegetation	*Quercetum rotundifoliae*	*Bupleuro rigidi‐Querceto rotundifoliae*	*Bupleuro gibraltarici‐Pistacieto lentisci*
Surrounding vegetation description	Close to riparian vegetation and diverse medium size shrubs and trees dominated by *Quercus rotundifolia*. It can be highlighted the presence of *Populus nigra, Acer monspessulanus, Cistus monspessulanum, Buxus sempervirens, Lonicera spp, Rosa canina, Thymus spp.,* and diverse Poacea*e* species	Riparian vegetation with prarie of grasses, diverse shrubs, close to *Quercus rotundifolia* formations and in transition with *Cephalanthero‐Quercetum faginae* vegetation. It highlights species such as *Salix spp, Populus nigra, Acer monspessulanum, Fraxinus angustifolia, Ficus carica, Crataegus monogyna*, *Juniperus oxycedrus*	Riparian vegetation, shrubs (e.g., *Retama sphaerocarpa, Nerium oleander, Chamaerops humilis, Pistacia lentiscus*) and small‐medium size trees such as *Ficus carica* or *Populus nigra*. Relative abundant presence of *Arundo donax* close to the river, and also diverse Poaceae species, especially in the plateau of the top of the cliffs. Existence of pinewood close to the climbing area
Notes of the land use/geo‐environmental resources	The area is within Moncayo Natural Park but the sampled cliffs were very close to the road (Clásica and Los Gaiteros at 50 m from the road and Espolón at 10 m from the road)	In a canyon forming a meadow through which a relatively narrow river runs. A dam has been established a few meters from the climbing area. Very popular area for climbing with many visitors	Relatively dry area very close to the road and to a river, which is sometimes dry in summer

## 2 MATERIALS AND METHODS

### 2.1 Study sites and field sampling

Soil samples from cliffs of three locations along Spain were collected in Spring 2021: Los Vados (Granada, South), Patones (Madrid, Centre), and Cálcena (Zaragoza, North) (Table 1). At each location, we sampled three climbing routes (Table 1). Specifically, to avoid potential climbers' interference, we collected soil samples from crevices more than 1 m distance at both sides of the climbing lines (i.e., the common ascension of the climbers guided by the parabolts and rope). To assess whether cliff specialist plant species differed in soil microbiota from control soils, we took from each route two soil samples where specialist cliff plants were growing and one sample from soils where plants were absent during the sampling (Figure 1). The crevices with soil including more than one cliff species were discarded. The specialist cliff plants associated with the sampled soils were *Pallenis maritima* L. (syn. *Asteriscus maritimus*; Asteraceae), *Chiliadenus glutinosus* (L.) Fourr. (syn. *Jasonia glutinosa*; Asteraceae), *Sedum dasyphyllum* L. (Crassulaceae), *Sarcocapnos enneaphylla* (L.) DC. (Papaveraceae), and *Cosentinia vellea* (Aiton) Tod. (Pteridaceae).

Soil samples were collected into 15‐ml Falcon tubes until they were filled, using a metal spoon, representing the majority of soil accumulated in the crevice. To collect soil, the first 1–3 mm were scratched away with the spoon to remove spores randomly present on the soil surface. Before scratching away the first 1–3 mm of the soil and before sampling each soil sample, the spoon was sterilized with Ethanol and then flamed. At the field site, soil samples were kept in a portable fridge and then stored in the laboratory at −20°C until DNA extraction.

### 2.2 Sequencing and amplicon‐metagenomics data analysis

For DNA extraction, we used 15 ml of soil and used a sieve (20 cm diameter and 0.25 mm mesh size) to grind the soil to fine powdery material. From this homogenized fine soil, we then took a sample of 250 mg for DNA extraction (Quick‐DNA Fecal/Soil Microbe Miniprep Kit; Zymo Research). Bead beating was run on a FastPrep‐24 instrument (MPBiomedicals; 4 cycles of 45 s at speed four followed by 1 cycle of 45 s at speed 6.5). A total of 400 μl of the raw extract was prepared for DNA isolation. The concentration of the isolated DNA was assessed with PicoGreen measurement (Quant‐iT™ PicoGreen™ dsDNA Assay Kit; Thermo Fisher).

To sequence the internal transcribed spacer (ITS2) regions of the fungal 18 S rRNA gene, two‐step, Nextera barcoded PCR libraries using the locus‐specific primer pair ITS3 (5′‐GCA TCG ATG AAG AAC GCA GC‐3′) and ITS4 (5′‐TCC TCC GCT TAT TGA TAT GC‐3′) with 20 PCR cycles for the first step and 20 PCR cycles for the second step were created. Subsequently, the PCR libraries were sequenced on an Illumina MiSeq platform using a v2 500 cycles kit.

The produced paired‐end reads which passed Illumina's chastity filter were subject to de‐multiplexing and trimming of Illumina adaptor residuals using Illumina's bcl2fastq software version v2.20.0.422. The quality of the reads was checked with the software FastQC version 0.11.8 (https://www.bioinformatics.babraham.ac.uk/projects/fastqc/), and sequencing reads that fell below an average Q‐score of 20 or had any uncalled bases (N) were removed from further analysis (for FastQC quality, *see* Figure S1). The locus‐specific V34 primers were trimmed from the sequencing reads with the software cutadapt v3.2 (Martin, 2011). Paired‐end reads were discarded if the primer could not be trimmed. Trimmed forward and reverse reads of each paired‐end read were merged to in silico reform the sequenced molecule considering a minimum overlap of 15 bases using the software USEARCH version 11.0.667 (Edgar, 2010). Merged reads containing ambiguous bases or outliers regarding the expected amplicon size distribution were also discarded. From the remaining reads, the fungal ITS2 subregions were extracted with the help of the ITSx software suite v1.1.3 and its included database (Bengtsson‐Palme et al., 2013).

The remaining reads were denoised using the UNOISE algorithm (Edgar, 2016) implemented in USEARCH to form operational taxonomic units (zOTUs—zero‐radius OTUs or ASVs—amplicon sequence variants), discarding singletons and chimeras in the process. The resulting ASV abundance table was then filtered for possible barcode bleed‐in contaminations using the UNCROSS algorithm (Edgar, 2018). ASV sequences were compared with the reference sequences of the UNITE database provided by https://www.drive5.com/usearch/manual/sintax_downloads.html, and taxonomies were predicted considering a minimum confidence threshold of 0.5 using the SINTAX algorithm implemented in USEARCH. The metagenome was visualized via krona charts (Ondov et al., 2011).

### 2.3 Statistical analysis

To describe the dominant lifestyles and diversity of fungi in cliff soils, we matched the trophic status information on the genus level to our dataset. For the trophic status information, we used a published lifestyle dataset (Tedersoo et al., 2014). We present the relative proportion of major taxonomic groups on order level for the diversity description. To roughly compare this relative proportion distribution against a background distribution, we gathered this information for Spain based on the GlobalFungi database (Větrovskỳ et al., 2020).

We used the estimated fungal ASV richness and Bray–Curtis community dissimilarity analyses to test for richness and composition differences among cliff locations. We calculated the estimated number of ASVs (ASV richness) based on R package *iNEXT* (Hsieh et al., 2016). For community dissimilarity analyses, we used the community matrix (ASV table). We first deleted local singletons (a cell with 1) and then used rarefaction. Rarefaction of the community matrix was done with the function rarefy of the R package *vegan* (Oksanen, 2015). Among available normalization methods, rarefaction was shown to be a reliable technique to remove differences in sampling effort (McKnight et al., 2019). We further square root transformed the community matrix before statistical analyses to down‐weigh samples with extremely high reads. The initial filtered community matrix (before standardization) had 342 ASVs and 999,569 reads.

To test whether fungal ASV richness and composition differ among locations and between soils with versus controls without cliff specialist plant, we used a liner mixed‐effect model (LMM) from the *lme4* package (Bates et al., 2015) and tested significance with an analysis of variance (ANOVA). The estimated fungal ASV richness was the response variable, and location, specialist plant presence (yes vs. no), and their two‐way interaction were the predictor variables. We further added the random effect climbing routes as an error term, which are the three replicates within each location. To test this model, we used the function *aov* in the R package *stats*. To test whether community dissimilarity differs among locations and with the presence of specialist plants, we used permutational multivariate analysis of variance using distance matrices (PERMANOVA). To fit this model, we used the function *adonis2* in the R package *vegan* with 999 permutations. As distance matrix, we used the Bray–Curtis dissimilarity matrix, and location, specialist plant presence, and their two‐way interaction as predictors.

Note that we further used a data subset to test for location differences. Our selected soils with a specialized cliff plant harbored different plant species. Thus, our analyses between soils of plant specialists and soil without plants, and also among locations, may be partly confounded by different plant species. We, therefore, added a subset analysis only based on the soil samples with the specialist cliff plant *S. dasyphyllum*, which was found and sampled repeatedly in the three study locations. This subset analysis allows testing the effect of the presence of specialist plants and location on fungal ASV richness and community composition independent from plant species diversity.

Finally, to characterize the location‐specific community better, we further performed an indicator species analysis based on the three locations using the function *indval* from the R package *labdsv* (Roberts, 2019). We listed all significant indicator ASVs that were available at the species level. Indicator species analysis can inform about species that are especially linked to a given habitat. The information of indicator species provides a set of species for easier monitoring of species change in the future (Dale & Beyeler, 2001).

## 3 RESULTS

**FIGURE 2:**
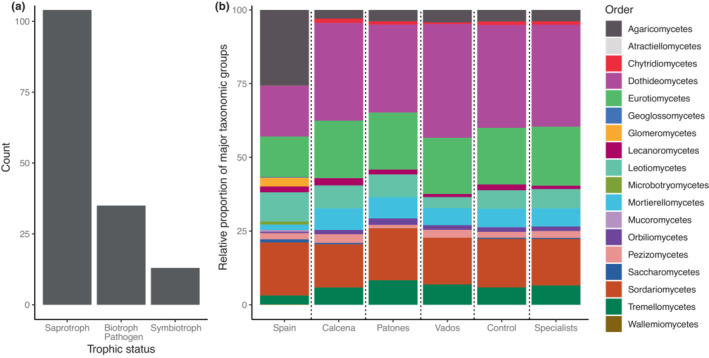
Relative proportion of sequences of trophy and major fungal orders. (a) Trophic status was coded based on Tedersoo et al. (2014) for 152 out of 342 ASVs based on genus information. For separate barplots for locations, see Table S2. (b) Cliff data are novel from this study. Data from Spain (on the left of the graph) were taken from Větrovskỳ et al. (2020) database.

We found a total of 342 ASVs after data processing. The ASVs detected are composed mainly of saprotrophic fungi, followed by biotrophs/pathogens and symbiotrophs (Figure 2a). Saprotrophic fungi were the main lifestyle across locations (Figure S2). Taxonomically, the ASVs belonged to the major fungal orders Dothideomycetes, Eurotiomycetes, and Sordariomycetes (Figure 2b). The five most abundant ASVs attributable at species level were *Cladosporium exasperatum* (Dothideomycetes), *Ulocladium chartarum* (Dothideomycetes), *Alternaria alternata* (Dothideomycetes), *Aureobasidium pullulans* (Dothideomycetes), and *Cryptococcus uzbekistanensis* (*Tremellomycetes*). To characterize more the fungal species present, we performed indicator species analyses. We found most indicator fungal species in Calcena, intermediate in Vados, while the smallest number was found in Patones. Symbiotroph species were identified in Calcena and Los Vados but not in Patones (for further location‐specific indicator species, *see* Table 2).

**TABLE 2:** Indicator species analysis for the three study sites. Only amplicon sequence variants (ASVs) listed, which were resolved to species level.

Location	Fungal species	Trophic status
Calcena	*Tulostoma brumale*	Saprotroph
	*Cryptococcus aerius*	Saprotroph
	*Mortierella exigua*	Saprotroph
	*Gibberella tricincta*	Biotroph
	*Gibberella baccata*	Biotroph
	*Lycoperdon lividum*	Saprotroph
	*Pseudogymnoascus destructans*	Saprotroph
	*Phomopsis velata*	Biotroph
	*Mortierella elongata*	Saprotroph
	*Gibberella tricincta*	Biotroph
	*Mortierella alpina*	Saprotroph
	*Mortierella exigua*	Saprotroph
	*Fusarium solani*	Biotroph
	*Filobasidium chernovii*	Saprotroph
	*Symbiotaphrina buchneri*	Symbiotroph
	*Penicillium armarii*	Saprotroph
	*Alternaria planifunda*	Biotroph
	*Geomyces auratus*	Saprotroph
	*Dothidea sambuci*	Saprotroph
	*Mortierella alpina*	Saprotroph
	*Penicillium jensenii*	Saprotroph
Patones	*Cladosporium exasperatum*	Saprotroph
	*Stemphylium herbarum*	Biotroph
	*Phaeosphaeria juncicola*	Saprotroph
	*Ulocladium chartarum*	Biotroph
	*Vishniacozyma victoriae*	Saprotroph
	*Acremonium acutatum*	Saprotroph
	*Acremonium charticola*	Saprotroph
Vados	*Arthrinium phaeospermum*	Biotroph
	*Camarosporium aloes*	Symbiotroph
	*Preussia terricola*	Saprotroph
	*Ganoderma resinaceum*	Biotroph
	*Lophiotrema rubi*	Saprotroph
	*Stemphylium herbarum*	Biotroph
	*Cryptococcus uzbekistanensis*	Saprotroph
	*Spencermartinsia plurivora*	Biotroph
	*Spizellomyces pseudodichotomus*	Saprotroph
	*Trichoderma effusum*	Biotroph
	*Phallus impudicus*	Saprotroph
	*Geastrum schweinitzii*	Saprotroph
	*Saitozyma flava*	Saprotroph
	*Botrytis cinerea*	Biotroph
	*Ulocladium chartarum*	Biotroph
	*Powellomyces hirtus*	Saprotroph
	*Acremonium persicinum*	Saprotroph

**FIGURE 3:**
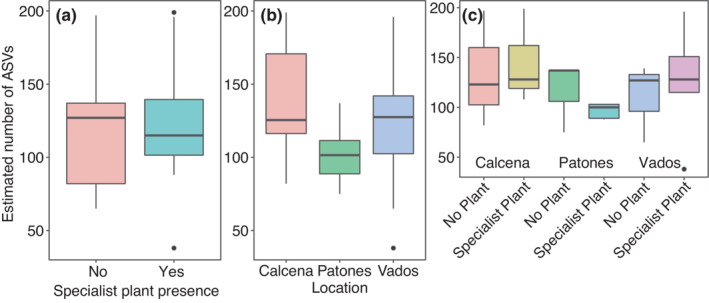
Estimated number of amplicon sequence variants (ASVs) found between soils with specialist plants and controls without plants (a), locations (b), and their interaction (c). For statistics table, *see* Table 3.

**FIGURE 4:**
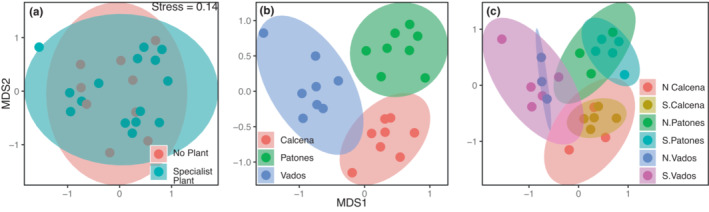
Ordinations based on nonmetric multidimensional scaling (NMDS) together with hulls for the presence of specialist plants in soil samples (a), location (b), and their interaction (c). In panel C, “S” represents soils of specialist cliff plants and “N” represents control soils with no plant. For statistics table, *see* Table 3.

We found no significant differences in ASV richness between soils with or without specialist cliff plants, among locations, or their two‐way interaction (Figure 3, Table 3). We found no significant differences in fungal community composition between soils with or without a specialist plant, but differences in fungal community composition were found among locations (Figure 4, Table 3). The interaction term of specialist plant presence and location was not significantly different for the community composition model. Note that we also found no significant effect of specialist plant presence on fungal ASV richness and marginal differences in fungal community composition (*F* = 1.74; *p* = .05) based on a data subset of the most abundant specialist cliff plant (*S. dasyphyllum*) against soils without plant (Table S1). In line with the overall samples, we found a significant effect of location on the fungal community composition when using the *S. dasyphyllum* data subset (Table S1).

**TABLE 3:** Statistical table for effects of the presence of specialist cliff plants in sampled soils (yes vs. no), location (i.e., Calcena, Patones, and Los Vados) and their interaction on ASV richness and community composition.

Predictor	ASV richness		Community composition		
	*F*	*p*	*R* ^2^	*F*	*p*
Specialist plant presence	2.92	.186	0.03	1.02	.386
Location	4.05	.141	**0.37**	**6.11**	**.001**
Specialist plant presence × Location	0.85	.512	0.06	0.94	.524

*Note*: For ASV richness, we used analysis of variance (ANOVA) with route as random term. For community composition, we fitted permutational multivariate analysis of variance using distance matrices (PERMANOVA). *F* statistics and *p*‐values are given, highlighting in bold the significant effects (*p* < .05) and for community composition additionally the partial coefficients of determination (*R* ^2^).

## 4 DISCUSSION

Cliffs are extreme environments with unique plant diversity, but our knowledge about microbes on cliffs in general and fungi‐plant syntrophic dependencies is still scarce. The ASVs found in our study sites are mainly Dothideomycetes and Eurotiomycetes, which occur in extreme environments (Coleine et al., 2021). Both groups form the majority of rock‐inhabiting fungi (RIFs), and one of the most abundant fungal species that we found was *Aureobasidium pullulans*, which is described as a typical rock‐inhabiting fungi species (Liu et al., 2022). Rock‐inhabiting fungi might thus play an important role in the recruitment of fungi from the bare rock into the soil and may help cliff plant development. To test this hypothesis, further studies should sample rocks in proximity to the cliff soils. We also compared the relative proportion of fungal taxonomic orders of our locations with samples from all over Spain (Figure 2). Based on this comparison, we found a relatively small proportion of Agaricomycetes within cliff soils. Agaricomycetes typically produce above‐ground sexual reproductive organs (e.g., mushrooms), which require the hyphae to build up a storage mycelium where glycogen is accumulated for later fruiting (Kües & Liu, 2000). Thus, in addition to the harsh abiotic conditions (e.g., intense solar radiation or high dryness) for optimal fruit bodies development, one potential explanation for the low proportion of Agaricomycetes might be the poorly developed soil of cliffs does not provide enough resources to support a storage mycelium.

Although locations did not significantly differ in fungal ASV richness, we found a significant effect of study location on fungal community composition, indicating a change in beta diversity in Spanish cliffs, and a noteworthy change in the described indicator species among localities, all showing a great variation in the fungal community. This, together with the high diversity found along with the cliff sites, suggests differences in their communities' functioning and roles in cliff habitats. Although fungi are supposed to have a little constraint in dispersal ability, it is often found that community composition differs already on relatively small spatial scales (Castaño et al., 2018; Krah et al., 2018). Our sampling locations are ca. 300 km, which is larger than the cited articles, but sampled cliff habitats were highly similar in many attributes (Table 1). The study locations have the same rock type (limestone) and neither differ substantially in soil chemical parameters (although a detailed assessment is still outstanding) nor in surrounding dominant vegetation. However, the sites differ in elevation and mean annual temperature parameters, which are known attributes to affect fungal diversity (Andrew et al., 2019; Diez et al., 2020; Tedersoo et al., 2014). Note that our dataset does not allow a multifactorial model including all these parameters. Further studies are needed to determine the mechanisms of the differences among the cliff locations.

Macro‐ and microclimatic differences are one important aspect to consider. Nonetheless, our study indicates that even in highly similar hostile habitats, there is a differentiation of fungal beta diversity, suggesting a high diversity of physiological adaptations to cope with the harsh conditions. Despite this, future studies should use culturing techniques to understand the physiological adaptations of cliff fungi better and investigate rocks for rock‐inhabiting fungi, which might be an important source of fungi in cliff soils. This exercise would allow a better understanding of the living and active fungal community and their abilities, which metabarcoding studies cannot accomplish. Finally, we found no significant differences in ASV richness among locations. One explanation might be that resource availability and energy are equally limited among locations by similar demographic rates. Thus, the richness found might be at the limit of carrying capacity in the three locations, leading to no substantial difference in richness. However, whether local harshness limits species richness to its carrying capacity is still not well understood (Hurlbert & Stegen, 2014; Marks et al., 2016).

Investigating particular fungal communities and species associated with specialist cliff plants is timely, since it was found that certain plants have developed specific symbiotic strategies with microorganisms to acquire soil nutrients where they are scarce (Augusto et al., 2019; Martin et al., 2017). There might be at least three explanations for our observation of no significant difference between soils with and without specialist plants. First, we found mainly saprotrophs, which are rather unspecific toward plant identity but rather substrate quality and plant polymer composition (Algora Gallardo et al., 2021). Further, the plant species present typically form arbuscular mycorrhiza with Glomeromycota species, lacking host specificity (Koide & Mosse, 2004). We further found biotrophs and pathogens, which are often host‐specific (King et al., 2011), but the harsh conditions may be a stronger selective factor than host specificity in cliff habitats. Second, our preliminary findings might result from statistical limitations (as we sampled more soils of specialist plants than control soils without plants, and thus the comparison is not balanced) or due to the presence of different plant species, which may hold different fungal diversity or composition. In fact, previous research found that plant diversity was a dominant factor affecting soil fungal community composition (He et al., 2017), and our analysis of *S. dasyphyllum* shows that fungal community composition tended to differ between soil with and without this specialist cliff plant. Thus, a greater sample size of selected plant species may reveal a clearer picture. This trend would be more in line with previous research identifying that functional diversity in fungi can influence the selectivity between plant and fungal partners (Chagnon et al., 2015) and may help to explain why specialist cliff plants can tolerate some human disturbances (March‐Salas et al., 2018). However, further in‐deep studies are needed to unravel whether specialist plants have developed certain adaptive and functional advantages thanks to microbiome associations, helping them cope with cliffs' stressful conditions. Third, another explanation might be that soils without plants were inhabited by other specialist plants in the years before the sampling, so soils are an artifact in terms of fungal diversity of early plant growth. However, the surface of soils with and without a specialist plant was, in most cases, visually different (personal observations). Soils with specialist plants frequently presented organic matter from previous plants colonization or from the same individual growing in the previous season, while soils without plants were mostly bare or partially covered by mosses, which were removed during the sample collection (as described in the “Materials and Methods” section). Thus, this alternative explanation may be less likely. The most abundant fungal trophic status found were saprotrophs, which decompose organic matter. For litter decomposing, fungi may select for specificity toward substrate composition rather than plant species‐specificity (Hättenschwiler et al., 2011). In this sense, our findings seem to be consistent with this latter view.

In conclusion, despite being extreme habitats, we found a high diversity of fungi in Spanish cliffs. Some species in cliff soils are known as rock‐inhabiting fungi and thus highly adapted species for life in this extreme habitat. Fungal communities differed among cliff locations in Spain, showing a change in beta diversity, but these preliminary results did not show great differences between soils with and without specialist plants. These findings thus suggest that cliffs harbor an important aspect of fungal diversity. Even within the cliff environment, there seems to be high variability in the involved fungal coping strategies. To better understand species strategies and biotic role with cliff plants, fungal isolates should be generated from soil and plants (e.g., from roots or seeds). Overall, our findings provide preliminary but novel information on fungal diversity and spatial variation in cliff environments and suggest new research routes to explore the existence of cliff plant‐specificity, and fungal functionality, and for testing patterns on fungal niche colonization and diversity.

## AUTHOR CONTRIBUTIONS

**Franz‐Sebastian Krah:** Conceptualization (equal); data curation (equal); formal analysis (lead); funding acquisition (equal); investigation (equal); visualization (lead); writing – original draft (lead); writing – review and editing (lead). **Marti March‐Salas:** Conceptualization (equal); formal analysis (supporting); funding acquisition (equal); investigation (equal); project administration (equal); writing – review and editing (supporting).

## FUNDING INFORMATION

Open Access funding enabled and organized by Projekt DEAL.

## CONFLICT OF INTEREST

None declared.

## Supporting information


Figure S1.

Figure S2.

Table S1.

## Data Availability

The datasets generated during and/or analyzed during the current study are available in the DRYAD repository (https://doi.org/10.5061/dryad.x95x69pp2).
