# Supplementary figures and images for: eDNA metabarcoding reveals high soil fungal diversity and variation in community composition among Spanish cliffs
Source: Ecol Evol. 2022 Dec 12;12(12):e9594. doi: 10.1002/ece3.9594 (PMC9745262; doi:10.1002/ece3.9594)

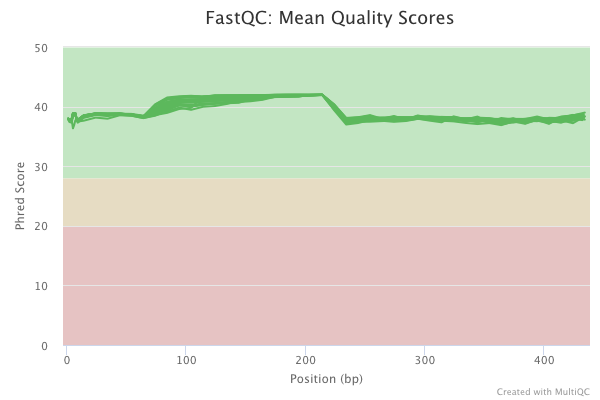

Supplement: Supplementary file 1 — Figure S1. [file ECE3-12-e9594-s001.tiff]

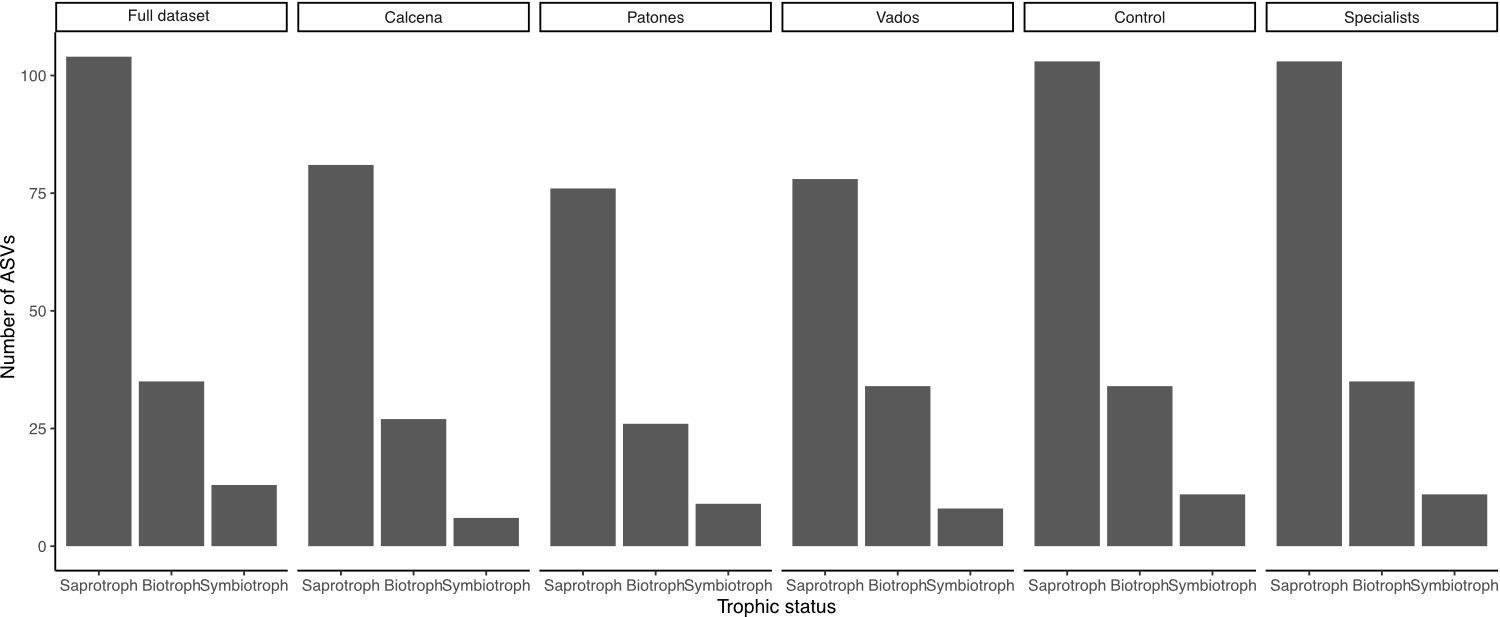

Supplement: Supplementary file 2 — Figure S2. [file ECE3-12-e9594-s003.jpg]
