# Supplementary material for: eDNA metabarcoding reveals high soil fungal diversity and variation in community composition among Spanish cliffs
Source: Ecol Evol. 2022 Dec 12;12(12):e9594. doi: 10.1002/ece3.9594 (PMC9745262; doi:10.1002/ece3.9594)
Supplement: Supplementary file 3 — Table S1. [file ECE3-12-e9594-s002.docx]

**Table S1.** Effect of the presence of specialist plant in soil samples and locations based on subset of data with *Sedum dasyphillum* (as the only cliff specialist occurring in all three locations). *F* statistics and *p*-values are given, highlighting in bold the significant effects (*p* < 0.05) and for community composition additionally the partial coefficients of determination (R^2^). We had to remove the interaction term due to data limitations.

|  | *ASV richness* | | *Community composition* | | |
| --- | --- | --- | --- | --- | --- |
| **Predictor** | ***F*** | ***P*** | **R^2^** | ***F*** | ***P*** |
| Specialist plant presence | 2.53 | 0.173 | 0.09 | 1.74 | 0.050 |
| Location | 0.35 | 0.722 | 0.38 | **3.54** | **0.001** |
